# Ventromedial prefrontal cortex, adding value to autobiographical memories

**DOI:** 10.1038/srep28630

**Published:** 2016-06-24

**Authors:** Wen-Jing Lin, Aidan J. Horner, Neil Burgess

**Affiliations:** 1 UCL Institute of Cognitive Neuroscience, 17 Queen Sq., London WC1N 3AZ, UK; 2 UCL Institute of Neurology, Queen Sq., London WC1N 3BG, UK; 3 Department of Psychology, University of York, York YO10 5DD, UK.

## Abstract

The medial prefrontal cortex (mPFC) has been consistently implicated in autobiographical memory recall and decision making. Its function in decision making tasks is believed to relate to value representation, but its function in autobiographical memory recall is not yet clear. We hypothesised that the mPFC represents the subjective value of elements during autobiographical memory retrieval. Using functional magnetic resonance imaging during an autobiographical memory recall task, we found that the blood oxygen level dependent (BOLD) signal in ventromedial prefrontal cortex (vmPFC) was parametrically modulated by the affective values of items in participants’ memories when they were recalling and evaluating these items. An unrelated modulation by the participant’s familiarity with the items was also observed. During retrieval of the event, the BOLD signal in the same region was modulated by the personal significance and emotional intensity of the memory, which was correlated with the values of the items within them. These results support the idea that vmPFC processes self-relevant information, and suggest that it is involved in representing the personal emotional values of the elements comprising autobiographical memories.

The medial prefrontal cortex (mPFC) has consistently been shown to play a role in autobiographical memory (AM) recall, for reviews, see refs 1, 2, 3, 4, recollection of self-relevant information^5^,^6^,^7^,^8^, the imagination of novel scenarios, for reviews, see refs 9 and 10, emotional regulation during autobiographical memory recall^11^,^12^ and linking self-relevance and value^13^,^14^, with the ventromedial prefrontal cortex (vmPFC), in particular, being reliably involved. Whilst this highlights that mPFC has an important role in AM and imagery, it remains unclear exactly what functional role it provides.

It is well-established that mPFC also plays a role in representing the values of choice during decision making, for a review, see ref. 15. In addition, judgements relating to the self are believed to be processed in more ventral mPFC while other-relevant processing is associated with more dorsal mPFC, for a review, see ref. 16. Taken together, these observations suggest that mPFC might contribute to imagination and AM by representing the subjective value of the contents of imagined or recollected scenarios, and that increasing the personal relevance of these contents might involve more ventral regions of mPFC.

The predicted modulation of mPFC activity by the value of elements within imagined scenarios has recently been observed^17^,^18^. In both studies, participants imagined novel scenarios and rated the subjective values of the imagined contents, with these ratings being found to correlate with activity in mPFC. Here we sought to investigate the hypothesis that activity in mPFC might reflect the values of elements of autobiographical memories, and the related hypothesis that the more personally relevant AMs used here might be reflected in activity in more ventral regions within mPFC than seen with the novel scenarios used in previous studies.

In our previous study^18^, activity in mPFC was modulated by participants’ subjective evaluation of common items present in newly imagined scenarios. In the present study, we used a similar procedure but replaced the imagined scenarios and imagined items with participants’ real autobiographical memories and the items that were remembered within them. On day1 we asked participants to recall AMs, including six items within each event that were either liked or disliked at the time of the event. They characterized each AM in terms of its pleasantness, recall vividness^19^, personal significance, recall frequency, recall difficulty, emotional intensity and time since it happened, and also reported the familiarity of the items at the time of the event (as familiarity has also been linked to mPFC activity during imagery^17^,^20^). On day 2, in an fMRI scanner, participants recalled AMs and then rated the values of four of the items in each event, and the vividness with which each was brought to mind.

## Methods

### Participants

Twenty-seven right-handed participants from University College London were recruited via advertisement. Two participants failed to finish the experiment and therefore all the results reported here were from the remaining 25 (11 males, mean age = 25.6, SD = 4.62, range = 20–35). All participants gave written informed consent before taking part in the experiment. All experimental protocols were approved by the UCL research ethics committee (1338/006), and all data collection and analyses were carried out in accordance with the approved guidelines.

### Stimuli

An AM interview procedure^21^ was adopted to collect participants’ autobiographical memories. Cue words used in the AM interview were 40 nouns chosen from Clark and Paivio’s^22^ extended norms. All of these words have high ratings in frequency (mean Thorndike-Lorge frequency = 1.88, SD = 0.15), imageability (mean = 6.32, SD = 0.39), and concreteness (mean = 6.59, SD = 0.55).

### Procedure

**Figure 1:**
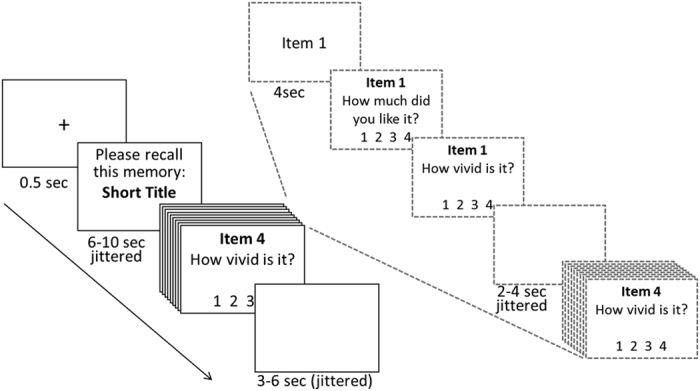
Day 2: Recall in scanner. Procedure of autobiographical memory recall and item rating in the scanner on Day2.

All participants took part in the experiment on two consecutive days–the AM interview on Day 1 and recall in the scanner on Day 2 (Fig. 1). Conducting a separate scanning session (on Day 2, as opposed to scanning the Day 1 interview) allowed us to have better control over several factors during memory and item retrieval in the scanner on Day 2, for instance, counterbalancing the order in which liked and disliked items were recalled and controlling the duration allowed for AM retrieval which can be extremely varied on Day 1. Although retrieval on Day 2 might be affected by the recall or rating process on Day 1^4^, participants were instructed to focus on their original memories. During the AM interview, all of the forty cue words were presented to participants one by one. Participants were instructed to freely associate one time- and location-specific autobiographical event to each cue word and verbally elaborate the details of the event. Details included the age of the event, location, people involved, and things that happened in the event. Events could range in age from their childhood to the day before the interview. A succinct memory title for each event was created by participants themselves as a reminder of the event to be used for recall on Day 2.

Ratings for each event were also required, including the pleasantness, recall vividness and emotional intensity evoked by the event, as well as its personal significance, recall frequency since the experience, and recall difficulty. These ratings are common in autobiographical memory studies, e.g., refs 23 and 24. Some of these ratings may be highly correlated with each other, for example, personal significance and emotional intensity. However, they were not identical, for example one participant had a highly positive affect when having delicious ice cream on a hot summer 10 years ago but this delicious-ice-cream memory did not have much personal significance. Participants also had to provide three items they liked and three they did not like from each event, as well as rating how familiar they were with each item at the moment when the event originally occurred. People could not be given as liked or disliked items. All the ratings in this study were on a scale from one to four. To give readers a better understanding of the types of event that were described, we present an example event from one of the participants:

‘The word “Journal” reminds me that I once stole my sister’s diary. This happened when I was 12, so that’s 2005 and it was December. My sister was keeping a diary since that summer but she never allowed me to read it. One day, I decided to steal it. It wasn’t so hard because I knew exactly where she hid it in our bedroom. I took her diary and sat on the floor next to my bed and began to read it. I liked the cover of the diary, it’s my favourite colour. In the first few pages, most of the contents were mundane things, so I got bored very soon. But I found one exciting page just before I wanted to stop reading - she was secretly in love with Orlando Bloom! She kept all the information about him and described how much she loves him. Just a few seconds after I found out this secret, I heard footsteps outside the room in the corridor. It was my sister. I was panicked and found no time for me to put the diary back in its place so I hid it under my duvet. She came into the room and realized what I was doing immediately. She got furious. We definitely had a very serious fight but I don’t really remember that part actually. So the three things I liked were the cover of that diary, the carpet on the floor I was sitting on, and my duvet. I didn’t like the drawer my sister hid her diary, the pair of shoes my sister was wearing and maybe the dome light in our room. I’m going to name this memory “Stealing my sister’s diary”.’

Although six items were obtained for each event on Day 1, only four were presented on Day 2–two liked and two disliked items. Only four items were used in the recall task to (1) shorten the duration that participants had to stay in the scanner and (2) avoid potential categorical differences between the ‘liked’ and ‘disliked’ items used for a given participant (e.g., avoiding all the liked items being snacks and all the disliked items being vegetables). The items used on Day 2 were selected by the experimenter and participants did not know which had been included until they saw them in the scanner. A liked item was chosen if (1) the same item or a very similar item had not been chosen yet, or (2) a similar item also existed among disliked items. The same principles applied when choosing disliked items.

During the recall task in the scanner on Day 2, participants recalled all forty memories and four of the items from each memory that they had provided on Day 1. There were two sessions in the recall task, each containing twenty trials. For each trial, participants were first required to retrieve the complete memory, followed by focussing their attention on specific items related to the memory. During memory retrieval, participants were instructed to reconstruct the scenario as closely as possible to the real situation when the event originally happened. They were encouraged to bring visual, auditory, tactile, olfactory and any other details into the reconstructed scenario. For instance, the beds, carpet, light, drawer and any other furniture in the bedroom, the setting of furniture, the sensation of holding the diary, the feeling of reading the diary, the sister’s handwriting, the sister’s footsteps and all the other details in the memory “Stealing my sister’s diary” should be reconstructed. While recalling and evaluating an item, attention should be focused on that item only. Specifically, participants were instructed to evaluate how much they liked this item and how vividly they recalled it.

Each trial consisted of the following sequence of stimuli: (1) a centrally presented fixation cross for 0.5 sec, (2) a title for memory retrieval, whose duration was randomly chosen between 6 to 10 seconds (uniform distribution), (3) a blank screen for 0.5 sec, (4) an item’s name for evaluation (item1) from the memory, presented for four sec, (5) ‘how much did you like item1?’ presented until a response was made (participants answered rating questions on a scale of 1 (not at all) to 4 (liked it very much) by pressing a button box with their right hands), (6) ‘how vivid is item1 now?’ presented until a response was made, (7) a blank screen for 2 to 4 sec (uniform distribution), steps (4–7) were repeated for the other three items from the event (i.e. item2, item3 and item4). The order of the presence of liked and disliked items was randomised across trials. A practice trial was carried out outside the scanner before participants went into the scanner.

Note that we asked participants to rate how much they liked the item during the event. However, in case their evaluation was influenced by their general liking for that type of item during daily life, after scanning we also asked them to give a rating of each item type used in the experiment (i.e., rating in general). For instance, if a participant had included book items, regardless of whether it was a statistics textbook, a science fiction novel, or a romance novel; they rated how much they liked books in daily life.

### fMRI Data acquisition and preprocessing

Functional imaging was performed on a 3T scanner (Siemens TIM Trio) during the autobiographical memory and item recall task. The functional data were acquired with a gradient-echo EPI sequence (TR, 3.36 s; TE, 30 ms; flip angle, 90°; resolution, 3 × 3 × 3 mm; 64 × 74; 48 slices per volume). The total number of volumes in each run varied across participants because of the variation of response time for each rating (the mean number of volumes was 329 per session, range = 248–468). A high-resolution T1-weighted 3-D structural image (1 mm3) was acquired after two sessions of functional scans. A double-echo FLASH fieldmap sequence was also recorded.

Functional images were processed and analysed with SPM8 (Wellcome Trust Centre for Neuroimaging, London UK, http://www.fil.ion.ucl.ac.uk/spm/software/spm8/). The first five volumes of each scan were discarded for T1 equilibration. Preprocessing procedures included bias correction, realignment, unwarping, coregistration, slice timing correction, and normalization to the MNI template using the Dartel toolbox. EPI images were smoothed with an isotropic 8 mm full-width half-maximum Gaussian kernel.

### Main analysis

The preprocessed functional images were analysed with general lineal models (GLMs). Along with regressors of interest, each GLM included 6 movement regressors for each session, estimated during realignment, as well as two further regressors modelling each session. Based on our strong a priori hypothesis that mPFC activity is modulated by the subjective value of memory content, we performed small-volume correction (SVC) within an anatomical mask of bilateral mPFC (volume ~53,493 mm^3^). This mask was derived from the AAL atlas^25^, as implemented in the WFU PickAtlas Tool^26^. Within this small volume we report effects that survive p < 0.05 family-wise error correction (FWE).

GLM1 was used for testing the hypothesis that mPFC represents value in autobiographical memory. According to our hypothesis, activity when recalling and evaluating liked items should be higher compared to recalling and evaluating disliked items. This model included five regressors per session: (1) recalling a memory, (2) evaluating a liked item from the memory (regardless of the item’s subjective rating; 2 items per memory), (3) evaluating a disliked item from the memory (regardless of the item’s subjective rating; 2 items per memory), (4) ITI periods and (5) key-presses. Trial periods were modelled with a boxcar function for the entire length of each period, convolved with the canonical HRF. Parameter estimates for regressors (1) to (3) were averaged across the two sessions and entered into a second-level model. The contrast between recalling a memory and evaluating an item (regardless of whether liked or disliked) was used to make sure participants were engaged in the AM recall task during scanning. We also compared the activity in mPFC when recalling a liked item versus recalling a disliked item.

GLM2 was used to further investigate the nature of the activity in mPFC found in GLM1 for liked versus disliked items, to see whether mPFC activity showed a parametric relationship to the subjective ratings of value given for each item. GLM2 included five regressors per session: (1) recalling a memory, (2) evaluating an item from the memory (regardless of liked/disliked), (3) a parametric modulator of the item regressor based on the participant’s value of that item (i.e., how much did they like this item within the event; a rating from 1–4), (4) ITI periods and (5) key-presses. A one-sample t-test was carried out in the 2^nd^ level analysis to test the effect of parametric modulator (regressor 3) averaged across the two sessions. Therefore, whereas GLM1 interrogates BOLD response for liked vs. disliked items (irrespective of their individual subjective rating in the scanner), GLM2 interrogates whether BOLD linearly varies with the individual subjective ratings of each item (irrespective of whether they are liked/disliked).

### Further analyses of vmPFC activity

We found significantly increased activity in vmPFC in GLM1 and GLM2 for objects with higher subjective value. However, we also found weak but significant correlations between the ratings of item value, and ratings of item recall vividness and of the item’s familiarity at the time of the event (see Results). Thus, these two factors might contribute to our observed item value effect. To investigate further, we also evaluated GLM3 within the vmPFC (an anatomical mask of bilateral vmPFC was derived from the AAL atlas, the volume ~15,513 mm^3^), which included 7 regressors, five of them were the same from those in GLM2, plus another two parametric modulators (PMs) – based on the recall vividness ratings and familiarity ratings of each item–in the following order: (1) recalling a memory, (2) evaluating an item, (3) vividness PM of regressor 2, (4) familiarity PM of regressor 2, (5) item value PM of regressor 2, (6) ITI, and (7) key-presses. Parameter estimates for regressor5 were averaged across the two sessions and entered into a second-level model (a one-sample t-test). In SPM, the first PM is allowed to explain both unique and shared variance, with subsequent PMs explaining the remaining unexplained variance of the preceding PMs. Thus, any value effects found in GLM3 is variance uniquely explained by the item value PM after removing shared variance from the preceding familiarity and vividness PMs.

Similarly, to assess any effects of item familiarity or item recall vividness independently from the other factor and from item value, we evaluated GLM4 and GLM5 with the PMs from GLM3 re-ordered so that familiarity and vividness came last respectively. Regressors in GLM4 were (1) recalling a memory, (2) evaluating an item, (3) vividness PM of regressor 2, (4) item value PM of regressor 2, (5) familiarity PM of regressor 2, (6) ITI, and (7) key-presses. Regressors in GLM5 were (1) recalling a memory, (2) evaluating an item, (3) familiarity PM of regressor 2, (4) item value PM of regressor 2, (5) vividness PM of regressor 2, (6) ITI, and (7) key-presses. Finally, to examine whether the event-specific item value effects we observed in GLM1 and 2 could reflect the values in everyday life of the types of item retrieved, we built GLM6. All the regressors and PMs in GLM6 were identical as those in GLM3 except that the last PM was the general value rating for that type of item in daily life. GLM6 was meant to detect any general preferences for different types of item that might modulate vmPFC activity. However, we note that this analysis of general preferences differs in nature from the analysis of the values of specific items.

To investigate the relation of the event-specific item value effects seen in vmPFC during the item evaluation phase to activity during retrieval of the corresponding autobiographical memory, we used additional GLMs for each of the ratings given to characterise the AMs in the initial meeting. Each GLM contained five regressors: (1) recalling a memory, (2) one of the memory rating PM of regressor 1, (3) evaluating an item, (4) ITI, and (5) key-presses. The PM regressor was one of the seven memory ratings, i.e. memory pleasantness, personal significance, recall frequency, recall difficulty, emotional intensity, recall detail, and memory age. Parameter estimates for regressor 2 were averaged across the two sessions and the percentage signal change in a 10-mm-radius region of interest (ROI) in vmPFC centred on the peak item rating effect in GLM3 (−6, +33, −12) was extracted by using MarsBaR toolbox^27^. A one-sample t-test was carried out for each GLM to test if there was any modulation of vmPFC ROI activity by one of the memory ratings when recalling memories.

### Behavioural results

#### Memory

The memory age ranged from one day to 31 years old. Table 1 shows the distribution of different memory ratings across all participants. Correlation coefficients between any two ratings are also present in Table 2.

#### Items

**Figure 2:**
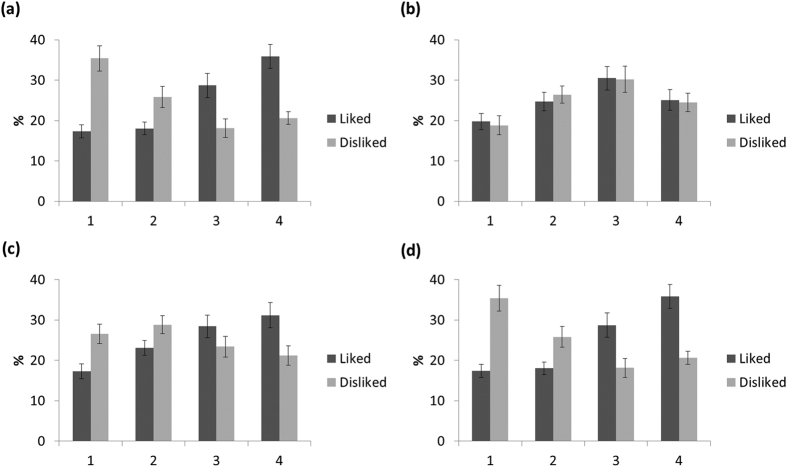
Percentage of liked and disliked items across ratings 1–4 on Day2. (**a**) Ratings for the specific items within an event that were initially identified as ‘liked’ or ‘disliked’ on Day1, showing higher ratings for the ‘liked’ items. (**b**) Ratings of participants’ preferences for these types of items in daily life, showing no differences between the categories of items from which the ‘liked’ or ‘disliked’ examples came. (**c**) Recall vividness for ‘liked’ and ‘disliked’ items within an event. (**d**) Familiarity rating of items (at the time of the event). Liked items were rated as more vividly recalled and more familiar at the time of the event than disliked items. Error bars represent ±1 SEM.

Only two liked and two disliked items from each memory were used in the scanning period on Day 2. Although some items appeared more than once across each participant’s reported memories, the influence of repetition should be negligible because the number of items was small (mean number of repeated items out of 160 used for each participant = 2.99, SD = 4.22). A two-way repeated measures ANOVA on rating of items was conducted to verify the value differences between liked and disliked items, including factors of event-specificity (i.e. value of that specific item within the event versus value of that type of item in general life) and item type (liked versus disliked). There were main effects of both rating specificity (F(1, 24) = 6.08, p = 0.021) and item type (F(1, 24) = 66.82, p < 0.001) and the interaction between them (F(1, 24) = 140.88, p < 0.001). Further analyses showed that ‘liked’ items had higher event-specific value ratings (t(24) = 13.17, p < 0.001) but not higher general value ratings (t(1, 24) < 1, p = 0.915). This suggests that the item value ratings in the scanner and the liked/disliked categorization prior to scanning do indeed reflect the event-specific value of the items concerned, not just the general values of these types of items in other circumstances. A paired-samples t-test on recall vividness between liked and disliked items revealed that liked items were more vivid than disliked items, t(24) = 10.85, p < 0.001. Figure 2 illustrates the value and recall vividness rating of items on Day 2.

Correlation coefficients (Spearman’s rank coefficient) between vividness rating and rating within events are listed in Table 3, as well as those between the familiarity rating and rating within events. In general, items with higher values tended to have both higher familiarity and vividness ratings.

### fMRI Results

#### Autobiographical memory recall

**Figure 3:**
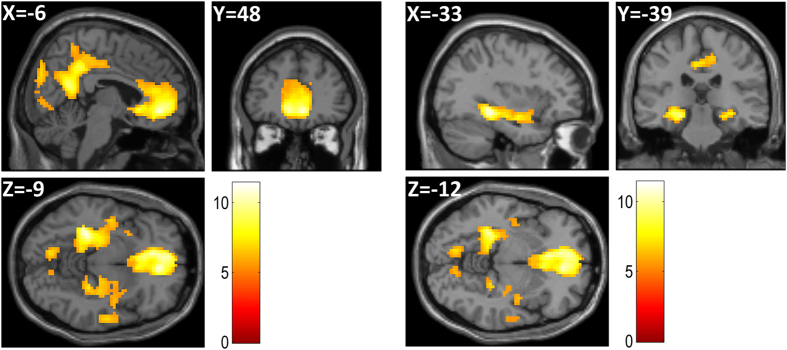
Autobiographical memory recall versus item memory. Plots shown at p < 0.005 FWE corrected, cluster size >1000 voxels.

We first searched for regions that showed a greater BOLD response when recalling a memory relative to evaluating an individual item (irrespective of liked/disliked) in GLM1, showing large regions of activity (p < 0.05, FWE) throughout the network that has consistently been associated with autobiographical memory recall, including mPFC, medial temporal lobes, retrosplenial and medial parietal areas (Maguire^21^; Svoboda *et al*.^3^) (Table 4 and Fig. 3).

#### Subjective value of items in mPFC

**Figure 4:**
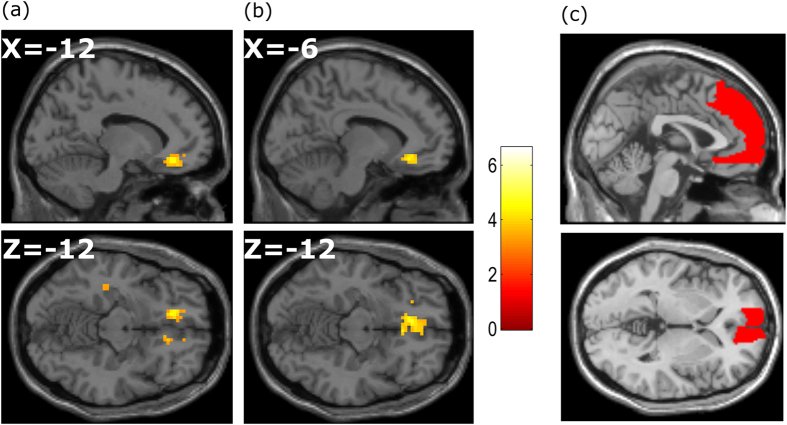
Item value effects. (**a**) The Liked- Disliked item contrast. (**b**) Parametric effect of subjective rating of value within event. Plots shown at p < 0.05 FWE, small volume corrected (SVC) using the anatomical mask of mPFC in (**c**).

To test our specific hypothesis, we first compared the evaluation of liked items to the evaluation of disliked items in GLM1 (liked > disliked). This contrast showed significantly greater activity in vmPFC (−12, +33, −12, Z = 4.23; p = 0.003 FWE SVC; Fig. 4). Furthermore, the parametric modulator of likability rating within event in GLM2 also revealed an effect in a similar area of vmPFC (−6, +33, −12, Z = 4.02; p = 0.008 FWE SVC, Fig. 4). In summary, we provide evidence that vmPFC shows greater activity for liked items and its activity positively correlates with the values of individual items from recalled autobiographical memories.

#### Relation of vmPFC value effects to familiarity and vividness

We found significantly increased activity in vmPFC in GLM1 and GLM2 for items with higher subjective value, consistent with our hypothesis. However, we also found weak but significant correlations between the ratings of item value, item recall vividness and the familiarity of the item at the time of the event (the latter from the initial interview), see Table 3. Thus, it is possible that these two factors might contribute to our observed item value effect in vmPFC. Accordingly, we examined vmPFC activity in more detail by including parametric modulators for item value, vividness and familiarity, rotating the order of parametric modulators across analyses (see Methods), and testing for significance within a mask focused on vmPFC using a SVC for this region. The value effect seen in vmPFC remained significant when familiarity and vividness were both included as parametric modulators in GLM3 (−6, +33, −12, Z = 3.58; p = 0.034 FWE SVC). These results support our hypothesis that vmPFC activity was modulated by the values of items in AMs, and that this effect cannot be fully explained by the familiarity of the item at the time of the event, or by the vividness of its recollection.

**Figure 5:**
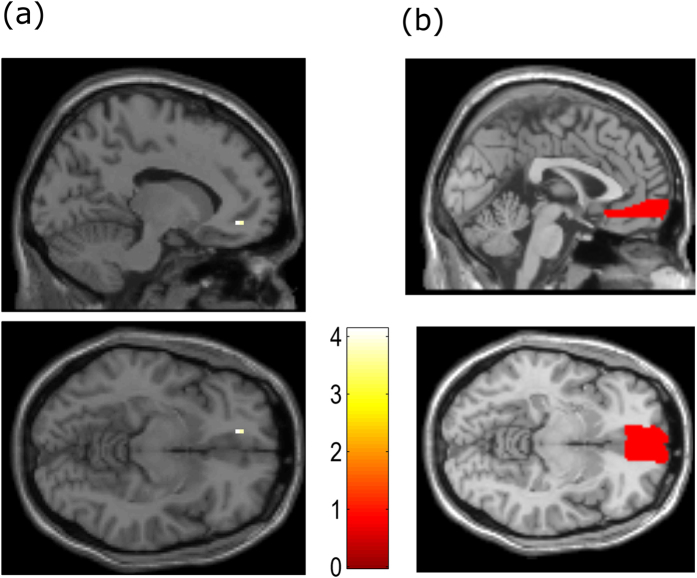
Effects of item familiarity. Effects of the familiarity of the item at the time of the event (**a**), Plots shown at p < 0.05 FWE, SVC on the vmPFC mask in (**b**).

The parametric modulator of item familiarity within event (GLM4) also revealed a significant unique effect in the vmPFC (−12, 42, −9, Z = 3.55; p = 0.043 FWE SVC; Fig. 5), which was not caused by the value or vividness of the items. This is in line with the account that vmPFC integrates affective value and familiarity of AM contents^17^. However, there was no unique effect of recall vividness within vmPFC (p > 0.05, Z = 2.83 FWE SVC) in GLM5, which suggests that the vividness of an item’s recall does not explain vmPFC activity beyond that explained by item familiarity or item value.

The involvement of vmPFC in value representation is well-known in decision-making tasks. However, there was no significant effect of the general every-day value of the types of items retrieved within vmPFC (GLM6). This suggests that the item value effect we observed in the present study reflected the memory-specific value of the item, rather than general preferences for different types of item. In sum, GLMs 3–6 suggest that, in our AM-focussed task, vmPFC independently tracks both the value and familiarity of the items within an event that is remembered in an autobiographical memory, rather than the non-specific values of these types of items in general.

#### Relation of vmPFC item value effects to the personal emotional significance of the memory

How might the subjective value of the items within an AM relate to processing during recall of the AM itself? The behavioural results show that there were significant correlations between the summed values of the items present in an AM and several of the ratings used to characterise that AM overall, including memory pleasantness, personal significance, recall frequency, recall difficulty, emotional intensity, recall detail, and memory age. We tested how activity in the vmPFC region showing the item value effect varied with these memory ratings during retrieval of the AM itself, using a separate GLM for each memory rating (see Methods). During recall of an AM, the mean activity in the vmPFC ROI varied with both the personal significance of the memory (p = 0.038) and the emotional intensity evoked by the AM (p = 0.0435). None of the other memory ratings showed significant modulation of vmPFC activity during recall of an AM.

It is likely that the subjective value of the items contribute to the personal emotional significance of the memories they occupy. This would explain the common response in this region to item value and to emotional intensity and personal significance. Example items include a birthday cake made by mom, a ticket to a favourite singer’s concert, a seashell collected from the beach during a family trip, rocks from grandfather’s collection, and a postcard from childhood friends.

## Discussion

Participants in an fMRI scanner recalled personal autobiographical memories (AMs), and evaluated their liking for specific items within each remembered event. Half of the items used were identified as ‘liked’ and the other half as ‘disliked’ within the context of each AM. Compared to ‘disliked’ items, the ‘liked’ items (in a specific event) were reported as being more familiar at the time of the event, and were recalled more vividly during memory recall. Consistent with the hypothesis that mPFC represents the value of items within AMs, vmPFC activity while recalling and evaluating items was modulated by how much participants liked those items at the time when the events happened.

During recall of the entire AM, activity in the vmPFC location showing the item value effect was modulated by the personal significance and the emotional intensity of the memory. This finding is in line with the ideas that vmPFC plays a role in the generation of affective meaning^28^, in the association of events with emotional responses^29^, and in the modulation of emotional response via self-relevance^30^. It is well recognised that the vmPFC is involved in self-relevant processing during autobiographical memory recall, e.g., refs 5 and 6. Consistent with the hypothesis by D’Argembeau that vmPFC assigns personal value to self-related information^31^, our results suggest that one of its roles is to provide the subjective values of the items present in AMs, and that these values contribute to the overall personal emotional significance of the AM itself.

In our previous study^18^, the activity of a region in mPFC (peak voxel coordinates: +9, +57, +12) was modulated by the subjective value of common everyday items that participants were imagining in novel scenarios. This region was more dorsal and anterior than the item value-related region in the present study (−6, +33, −12). A functional gradient along dorsal-ventral axis has been observed in mPFC, between making self- or other-related judgements, such that self-relevant information is believed to be processed in more ventral mPFC, whereas other-relevant information is processed in more dorsal mPFC^16^,^32^,^33^. Similarly, vmPFC activity during memory for recent presentation of face stimuli is greater for personally relevant faces^34^. Thus the more ventral location of the item value effect here, compared to Lin *et al*.^18^, may reflect the greater personal emotional relevance of the items from participants’ autobiographical memories compared to the photos of common everyday objects used in the previous study.

Speer *et al*.^35^ discovered that mPFC activity was greater during the recall of AMs that made them feel happy compared to the recall of neutral memories, using a similar paradigm to our own. In their study, ventral striatum activity was also parametrically modulated by affective ratings of the memories. Both ventral striatum and mPFC also responded to monetary reward in their study, and participants were even willing to lose monetary reward to obtain chances to recall positive memories. Speer *et al*.^35^ suggested that positive memory recall is valuable, so that the reward system was recruited in positive memory recall. Compared to Speer *et al*.^35^, our study indicates that vmPFC can represent the values of different memory components separately, i.e., the items within memories. We also noticed that, during recall of an AM, vmPFC activity was modulated by the personal significance and emotional intensity of the AM, implying that the variation in vmPFC activity with the subjective value of items relates to the part those items play in the emotional self-relevance of the event. If the value-related vmPFC effect in our study reflects items’ personal emotional relevance, this might explain the reduced involvement of ventral striatum (+12, +3, −3, p = 0.25) here compared to Speer *et al*.^35^, assuming that ventral striatal activity reflects the subjective consequences of recalling a positive memory (i.e. the feeling of happiness) which has direct value for the current state (equivalent to receiving money), consistent with its association with reward magnitude more generally^36^,^37^,^38^,^39^,^40^.

In addition to subjective value, greater levels of activity in vmPFC have also been reported when participants recalled a familiar memory or imagined personal future events within a familiar contextual setting, compared to imagining personal future events within an unfamiliar contextual setting^17^,^20^. Consistent with the results from these studies, vmPFC activity was also modulated by how familiar the items were at the time of the AM in our study, being greater when recalling and evaluating more familiar items. However, the unique effects of item value and item familiarity occur independently in vmPFC, and there were no significant correlation between a memory’s personal significance or emotional intensity and the summed familiarity of the items within it. Further studies are necessary to clarify the nature and importance of item familiarity in modulating vmPFC activity.

In summary, we showed that vmPFC activity is modulated by the values of items within autobiographical memories. Taken together with our previous study^18^, these results are consistent with the hypothesis that mPFC represents the values of elements within autobiographical memory and mental imagery, with the more ventral mPFC location found in the present study reflecting the greater emotional self-relevance of objects in autobiographical memories than those in arbitrary imagined scenarios. In addition, our findings support the association of vmPFC activity with processing of self-relevance and, in our study, with the contribution of liked objects to the personal emotional relevance of autobiographical memories.

## Additional Information

**How to cite this article**: Lin, W.-J. *et al*. Ventromedial prefrontal cortex, adding value to autobiographical memories. *Sci. Rep.* **6**, 28630; doi: 10.1038/srep28630 (2016).

## Figures and Tables

**Table 1:** The results of memory ratings.

	1	2	3	4	Mean
How much did you like this event? (1 not at all-4 very much)	17%	18%	31%	33%	2.81
Level of detail? (1 vague-4 vivid)	11%	31%	36%	22%	2.69
Emotional intensity evoked by the memory? (1 non-emotional-4 highly-emotional)	17%	38%	31%	14%	2.41
Personal significance of this memory? (1 insignificance-4 life-changing)	27%	36%	27%	10%	2.20
How often do you recall this memory?(1 never-4 very often)	34%	45%	18%	4%	1.92
Difficulty of recall? (1 very easy-4 very difficult)	36%	42%	17%	5%	1.91

**Table 2:** Mean Spearman’s rank correlation coefficients (rho) between memory ratings.

	(1)	(2)	(3)	(4)	(5)	(6)	(7)
(1)pleasantness	–						
(2)detail	0.255^***^	–					
(3)emotional intensity	0.149^**^	0.312	–				
(4)significance	0.195^***^	0.268^***^	0.602^***^	–			
(5)recall frequency	0.143^***^	0.406^***^	0.371^***^	0.423^***^	–		
(6)recall difficulty	−0.049	−0.491^***^	−0.139^**^	−0.159^**^	−0.369^***^	–	
(7)memory age	−0.059	−0.422^***^	0.064	0.102^*^	−0.115^*^	−0.407^***^	–

*Significant correlation at p < 0.05.

**Significant correlation at p < 0.01.

***Significant correlation at p < 0.001.

**Table 3:** Mean Spearman’s rank correlation coefficients (rho) between item ratings.

	(1)	(2)	(3)
(1)rating within event	–		
(2)recall vividness	0.238^***^	–	
(3)familiarity	0.151^***^	0.221^***^	–

***Significant correlation at p < 0.001.

**Table 4:** Results of the contrast comparing autobiographical memory recall to item recall.

Region	Cluster Size	x	y	z	Peak	Peak
					T	p_(FWE-corr)_
ventromedial Prefrontal Cortex	1660	6	42	−9	10.73	<0.001
Inferior Frontal Gyrus	44	−30	33	−9	7.46	<0.001
Middle Frontal Gyrus	103	24	30	39	7.15	<0.001
Inferior Frontal Gyrus	35	48	30	9	6.81	<0.001
Sub-Gyral	169	−21	27	39	8.98	<0.001
Superior Temporal Pole	12	42	24	−24	5.22	0.016
Superior Temporal Gyrus	12	51	12	−9	5.83	0.002
Superior Temporal Gyrus	383	42	−54	21	9.02	<0.001
Middle Temporal Gyrus	69	−54	−54	−6	6.26	<0.001
Posterior Cingulate	4024	−9	−57	24	11.40	<0.001
Middle Temporal Gyrus	296	−42	−69	24	9.33	<0.001
Cerebellum	18	12	−45	−42	6.49	<0.001
Cerebellar Tonsil	16	−9	−48	−45	5.55	0.005
